# Acetoacetate is a more efficient energy-yielding substrate for human mesenchymal stem cells than glucose and generates fewer reactive oxygen species

**DOI:** 10.1016/j.biocel.2017.05.007

**Published:** 2017-07

**Authors:** Mary Board, Colleen Lopez, Christian van den Bos, Richard Callaghan, Kieran Clarke, Carolyn Carr

**Affiliations:** a Department of Physiology, Anatomy and Genetics, University of Oxford, South Parks Road, Oxford OX1 3 PG, United Kingdom; b California State University, San Marcos, USA; c Mares Ltd., Muensterstrasse 87, 48268 Greven, Germany; d Research School of Biology, ANU College of Medicine, Biology and the Environment, Australian National University, Canberra, Australia

**Keywords:** hMS, Chuman mesenchymal stem cells, p2, passage 2, p5, passage 5, Acetoacetate, Glycolysis, Oxidation, ROS, Stem cells

## Abstract

Stem cells have been assumed to demonstrate a reliance on anaerobic energy generation, suited to their hypoxic *in vivo* environment. However, we found that human mesenchymal stem cells (hMSCs) have an active oxidative metabolism with a range of substrates. More ATP was consistently produced from substrate oxidation than glycolysis by cultured hMSCs. Strong substrate preferences were shown with the ketone body, acetoacetate, being oxidised at up to 35 times the rate of glucose. ROS-generation was 45-fold lower during acetoacetate oxidation compared with glucose and substrate preference may be an adaptation to reduce oxidative stress. The UCP2 inhibitor, genipin, increased ROS production with either acetoacetate or glucose by 2-fold, indicating a role for UCP2 in suppressing ROS production. Addition of pyruvate stimulated acetoacetate oxidation and this combination increased ATP production 27-fold, compared with glucose alone, which has implications for growth medium composition. Oxygen tension during culture affected metabolism by hMSCs. Between passages 2 and 5, rates of both glycolysis and substrate-oxidation increased at least 2-fold for normoxic (20% O_2_)- but not hypoxic (5% O_2_)-cultured hMSCs, despite declining growth rates and no detectable signs of differentiation. Culture of the cells with 3-hydroxybutyrate abolished the increased rates of these pathways. These findings have implications for stem cell therapy, which necessarily involves *in vitro* culture of cells, since low passage number normoxic cultured stem cells show metabolic adaptations without detectable changes in stem-like status.

## Highlights

• Human mesenchymal stem cells oxidise acetoacetate 35 times faster than glucose.
• Oxidation of acetoacetate reduces ROS-production 45-fold compared with glucose.
• Acetoacetate plus pyruvate is the optimal substrate combination for ATP-production.
• Culture of hMSCs in 20% oxygen upregulates energy metabolism 2-fold.

## Graphical abstract

**Energy yielding pathways of human mesenchymal stem cells**

Glucose is consumed by both the glycolytic and the oxidative pathways. Pyruvate, acetoacetate and glutamine are oxidised by hMSCs. Exogenous substrates are shown in boxes with solid lines, pathway intermediates in boxes with dashed lines and products in boxes with dotted lines. Arrows showing routes of entry of exogenous substrates into pathways are weighted to represent rates of utilisation (not to scale).

**Figure:**
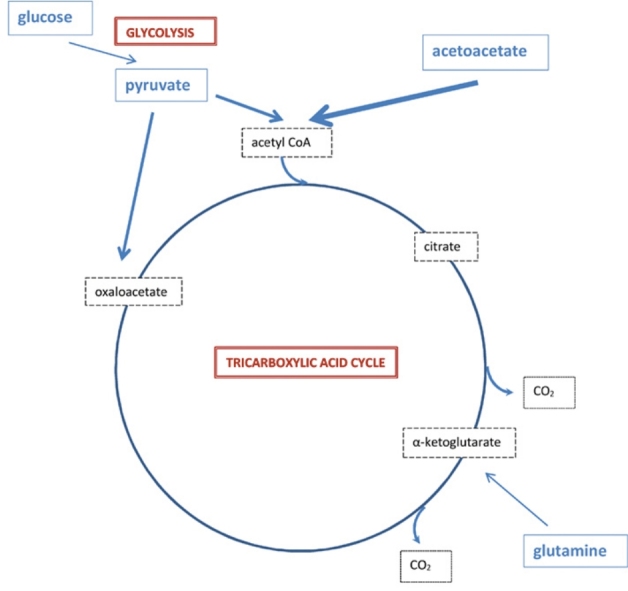

## 1 Introduction

The potential for stem cell therapy has received a great deal of popular and scientific attention over recent years with proposals that transplantation of cells could take the form of an acute treatment for myocardial infarction, for example, or a chronic treatment for neurodegenerative disorders. Any form of therapy involves the extraction, storage and *in vitro* culture of cells raising the possibility that these processes (*in vitro* culture, in particular) would have an impact on the characteristics of the cells. Cytoskeletal and other phenotypic changes have been reported for mesenchymal stem cells in long-term culture of 40 passages (Vacanti et al., 2005) and this field has been reviewed by Bara et al. (2014). There is also the possibility that changes to the cells’ energy metabolism occur and the present study investigates some aspects of this, allowing presentation of results indicating substrate selection and energy generation by human mesenchymal stem cells (hMSCs) under conditions of normoxic and physiologically relevant hypoxic culture between passages 2 and 5.

Cells in culture are normally exposed to glucose and glutamine and, occasionally, pyruvate in their growth media but a wider range of energy yielding substrates may be available to the cells *in vivo*. Plasma ketone body levels may reach or exceed 7 mM under conditions of starvation or a ketogenic diet, compared with normal levels in the fed state of around 0.1 mM (Hall et al., 1984). With ketone body levels of 7 mM, it may be that this species will be utilised by the stem cells to generate energy. Indeed, there are indications that stem cells *in vivo* may benefit from higher concentrations of ketone bodies which may influence their capacity to contribute to neurogenesis, among other processes (Lee et al., 2002a, Lee et al., 2002b). The potential contribution made to hMSC energy metabolism by the consumption of ketone bodies was investigated during the present study.

It is considered that mesenchymal stem cells *in vivo* occupy a hypoxic niche (Schofield, 1978, Crisan et al., 2008, Parmar et al., 2007, Suda et al., 2011) and that their energy yielding metabolism is likely to be correspondingly hypoxic and to include a reliance on the pathway of glycolysis for ATP generation (Pattappa et al., 2011, Simsek et al., 2010; reviewed by Gaspar et al., 2014). Much has been written about the relationship between hypoxia and differentiation of stem cells and suggestions have been made as to the adaptation and maintenance of hypoxic metabolism in these cells, including that they have immature and dysfunctional mitochondria (reviewed by Xu et al., 2013). We have assessed the contribution made to energy metabolism by the anaerobic glycolytic pathway and by the oxidative metabolism of hMSCs cultured *in vitro* under conditions of hypoxia (5% O_2_) and normoxia (20% O_2_). It is anticipated that stem cells would have a large ATP turnover to sustain, since these cells show a high rate of proliferation. (It has been estimated that the production of a single daughter *E.coli* cell accounts for the consumption of 2.36-2.9 × 10^13^ molecules of ATP (Hempfling and Mainzer, 1975, Farmer and Jones, 1975). The present study analysed a range of potential energy yielding substrates and calculated the contribution made to ATP synthesis in hMSCs growing in culture. Both the glucose and glutamine routinely included in growth medium can be used by these cells to generate ATP and pyruvate, where present, may also fulfil this role. The current study demonstrates the contributions made by these substrates and by the ketone body, acetoacetate, to ATP generation. The analysis represents a way of indicating which are potentially the most effective energy yielding substrates and thus what modifications to stem cell growth medium might be advantageous. The findings may also shed light on beneficial *in vivo* conditions for proliferation of stem cells in order to facilitate putative stem cell therapy.

Recent reports have indicated that the *in vitro* or *in vivo* consumption of ketone bodies by cells, particularly neurones, may represent an adaptation to alleviate oxidative stress (Fukao et al., 2004, Maalouf et al., 2009, Shimazu et al., 2013). Given the suggestions that stem cells reside in hypoxic niches *in vivo* partly to avoid generation of ROS (Suda et al., 2011), it may be that this cell-type would have a preference for a low ROS-generating substrate. Thus, we have measured rates of production of ROS by hMSCs after exposure to glucose and acetoacetate and assessed the effects of the UCP2-inhibitor, genipin, on production of ROS with different substrates.

## 2 Materials and methods

All consumables were purchased from Sigma Aldrich unless otherwise indicated.

### 2.1 Human mesenchymal stem cells

Stem cells were obtained from 3 sources: generous gifts from Lonza and Dr. Yasser El-Sherbini, Dept. of Biomedical Engineering, University of Oxford and purchased from Millipore.

#### 2.1.1 Culture of cells

Cryopreserved cells were thawed, washed with growth medium and seeded at a density of 1 × 10^6^ cells per 225 cm^2^ with DMEM containing 2 mM ultra-glutamine, 5.5 mM glucose, 50 μg/ml gentamycin and 10% FBS (Lonza). Where 3-hydroxybutyrate (3-HB) was included in the growth medium, the concentration was 5 mM throughout. Cells were passaged at about 80% confluence. Cells for measurements of glycolysis or oxidation were seeded into 24-well plates at a density of 1 × 10^4^ cells per well and used at 90% confluence. Cells were maintained in either normoxic culture with 20% O_2_ tension or hypoxic culture with 5% O_2_ tension in an oxygen tension controlled incubator. Previous workers have identified 5% O_2_ as being a physiologically significant level of O_2_ with respect to *in vivo* conditions for hMSCs, representing hypoxia (Pattappa et al., 2011).

### 2.2 Measurements of metabolic flux

#### 2.2.1 Measurements of substrate-oxidation in 24-well plates

Cells were incubated for 90 min in the presence of a single radiolabelled substrate (with addition of unlabelled substrates where indicated) in DMEM (containing no pyruvate, glucose or glutamine) in a total volume of 0.5 mls. The concentrations of single substrates were: 5.5 mM glucose (considered similar to median human blood glucose concentration); 2 mM pyruvate (considered higher than endogenous pyruvate concentration from glucose metabolism and sufficient to stimulate activity of pyruvate dehydrogenase plus pyruvate carboxylase); 1 mM glutamine (considered similar to human blood glutamine concentrations); 10 mM acetoacetate (considered similar to intracellular acetoacetate concentrations produced from maximal blood ketone body concentrations). In addition, substrates incorporated radiolabels as follows: glucose: 21 MBq/mmol D-U-^14^C-glucose (Perkin Elmer); pyruvate: 0.35MBq/mmol 1-^14^C-pyruvate (American Radiochemicals); acetoacetate: 0.185MBq/mmol 3-^14^C-acetoacetate (American Radiochemicals); glutamine: 1.2 MBq/mmol U-^14^C-glutamine (Perkin Elmer). Evolved ^14^CO_2_ was trapped and measured by the method of Collins et al. (1998), except that, after addition of perchloric acid to the wells, plates were gently agitated for 60 min to release dissolved ^14^CO_2_. Filter papers containing trapped ^14^CO_2_ were counted in Ecoscint (National Diagnostics) using a Tri-Carb 2800TR Liquid Scintillation Analyser. Preliminary measurements showed that rates of ^14^CO_2_-production were linear for a period of at least 120 min under these conditions. Rates of ATP-production from individual substrates were calculated on the basis of mol/mol production as follows: glucose: 31; glutamine: 20; pyruvate: 12.5; acetoacetate: 20. For calculations of ATP-production from acetoacetate plus pyruvate, separate incubations were performed with ^14^C-acetoacetate in the presence of unlabelled pyruvate and with ^14^C-pyruvate in the presence of unlabelled acetoacetate. Rates of ATP-production from the labelled substrate were calculated for each condition and the results summed.

#### 2.2.2 Measurements of glycolytic rate in 24 well plates

Glycolysis was measured as the production of ^3^H_2_O from 5-^3^H-glucose, which occurs during the step catalysed by enolase. Cells were incubated with 5.5 mM glucose containing 17 MBq/mmol 5-^3^H-glucose (Perkin Elmer) in DMEM without pyruvate, glutamine or glucose for 90 min in a total volume of 0.5mls. At 90 min, 0.2 mls of the medium was applied to a column (volume 1 ml) of Dowex-1-borate (prepared from Dowex-1-chloride by the method of Hammerstedt, 1973). Washing with 2 column volumes of water eluted the ^3^H_2_O which was counted for radioactivity (Tri-Carb 2800TR Liquid Scintillation Analyser).

#### 2.2.3 Calculation of ATP yield

ATP yields were calculated from the following values: from glycolysis of glucose, it is assumed that 2 mol ATP will be produced per mole glucose glycolysed. For substrate oxidation measurements, it is assumed that full oxidation of each substrate takes place. Under these conditions, glucose oxidation will produce a mean value of 31 mol ATP/mol glucose when glucose is converted to CO_2_ by the action of glycolysis, the pyruvate dehydrogenase reaction and reactions of the tricarboxylic acid cycle. It is assumed that cytosolic NADH generated from glycolysis will be re-oxidised due to the action of either the 3-phosphoglycerate or the malate-aspartate shuttles. Pyruvate oxidation generates 12.5 mol ATP/mol pyruvate when the substrate is fully converted to CO_2_ by the actions of the pyruvate dehydrogenase reaction and reactions of the tricarboxylic acid cycle. Acetoacetate oxidation generates 20 mol ATP/mol acetoacetate when the substrate is first split into acetyl coenzyme A which is fully converted to CO_2_ by reactions of the tricarboxylic acid cycle. Glutamine oxidation generates 20 mol ATP/mol glutamine when the substrate is deaminated to α-ketoglutarate and α-ketoglutarate converted to pyruvate by reactions of the tricarboxylic acid cycle plus that of pyruvate carboxylase. The resulting pyruvate is then oxidised in the same way as exogenous pyruvate.

#### 2.2.4 Measurement of reactive oxygen species

Cells were incubated under appropriate conditions in 24-well plates in a total volume of 0.2 ml for 90 min. At 90 min, ROS were assayed using an Oxiselect Amplex Red kit (Cell Biolabs, Inc.) according to the manufacturer’s instructions. Where genipin was used, cells were pre-incubated for 24 h with 20 μM genipin.

### 2.3 Growth and differentiation experiments

#### 2.3.1 Growth rates of hMSCs

At each passage (typically every 48 h), cell numbers were counted in a haemocytometer using Trypan Blue exclusion as a gauge of viability.

Please see Supplementary Information for measurements of mRNA production of marker proteins and differentiation of hMSCs into osteocytes, adipocytes and chondrocytes (Methods and Data).

### 2.4 Detection of UCP2 by western blotting

Western blots were performed under denaturing conditions. Cells were detached using trypsin, homogenised using a 21G needle, incubated for 5 min in a dri-block at 100 °C and centrifuged at 13,000 rpm for 10 min. Aliquots of the supernatant were assayed for protein concentration (Pierce BCA protein assay kit) and the remainder had 5% mercaptoethanol added before incubation for 5 min at 100 °C. Blots were performed by the method of Heather et al. (2011), where equal quantities of total cellular protein (25 μg) were loaded per well, proteins were stained with Ponceau’s reagent and gels blocked with 5% milk powder. Loading of wells with equal amounts of protein allows normalisation of expression for comparative purposes and avoids the possibility that expression of housekeeping genes may change with passage number or with cell treatment protocol (Farmer et al., 1983, Gorr and Vogel, 2015). UCP2 protein was detected using rabbit polyclonal anti-human UCP2 antibody (5 μg/ml) purchased from Abcam and donkey anti-rabbit secondary antibody (to give a ratio of secondary: primary antibody of 1.6) allowed detection by exposure to X-ray film. Rainbow marker proteins (25–120 kDa) were used to assess molecular weight.

### 2.5 Data analysis

**Fig. 1:**
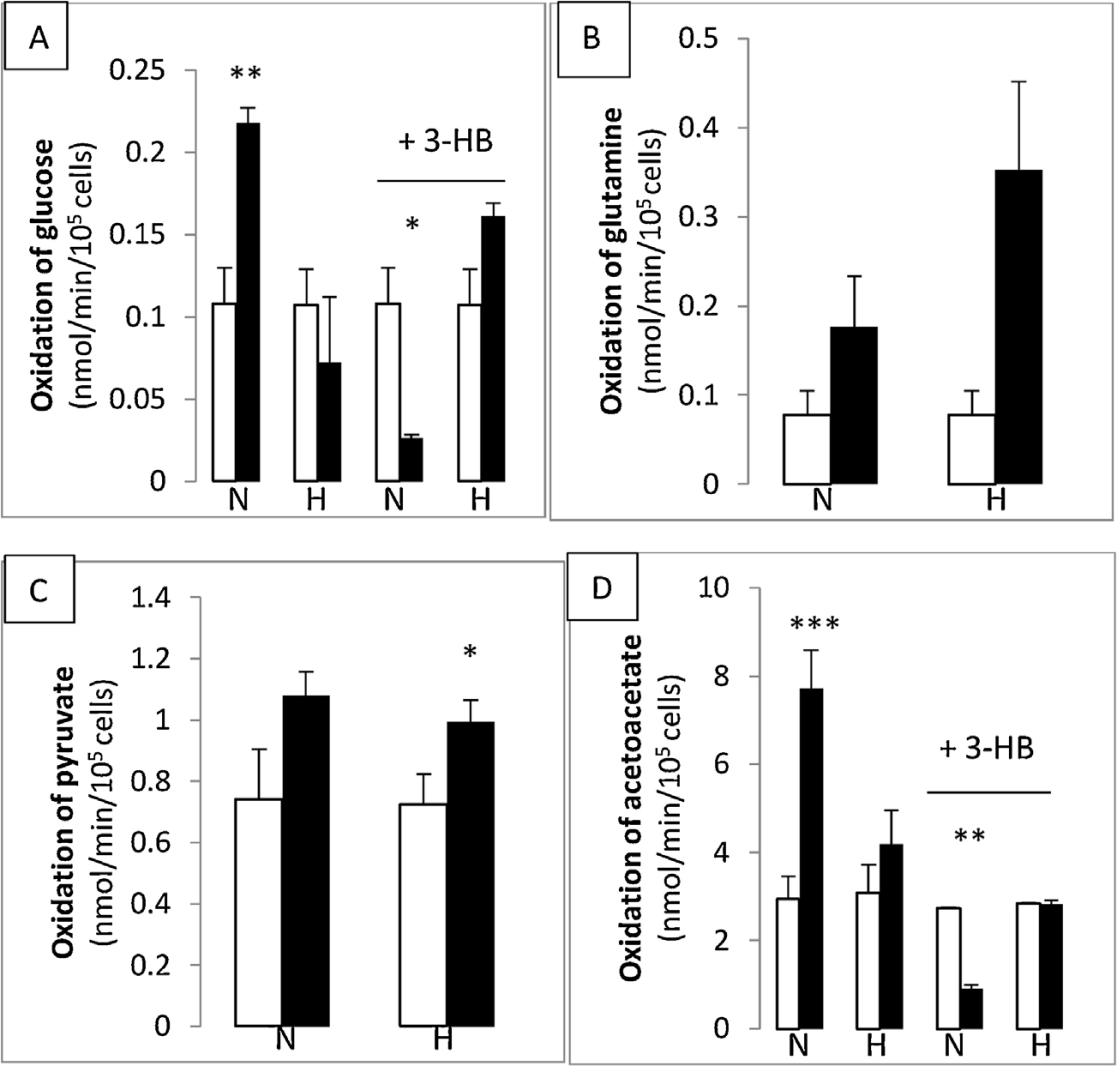
Oxidation of energy yielding substrates by hMSCs. Rates of oxidation of 5.5 mM glucose (A); 1 mM glutamine (B); 2 mM pyruvate (C) and 10 mM acetoacetate (D) are shown for cells at passage 2 (open columns) and passage 5 (solid columns). Rates of substrate oxidation were measured in 24 well plates as the production of ^14^CO_2_ from ^14^C-labelled substrates. Cells were cultured as follows through passages 1–5: normoxia (20% O_2_, N) or hypoxia (5% O_2_, H) with 5 mM 3-HB, where indicated. n ≥ 5 for each column. *: p < 0.05, ** p < 0.01 and ***p < 0.0001 when substrate oxidation at p5 is compared with that at p2.

**Fig. 2:**
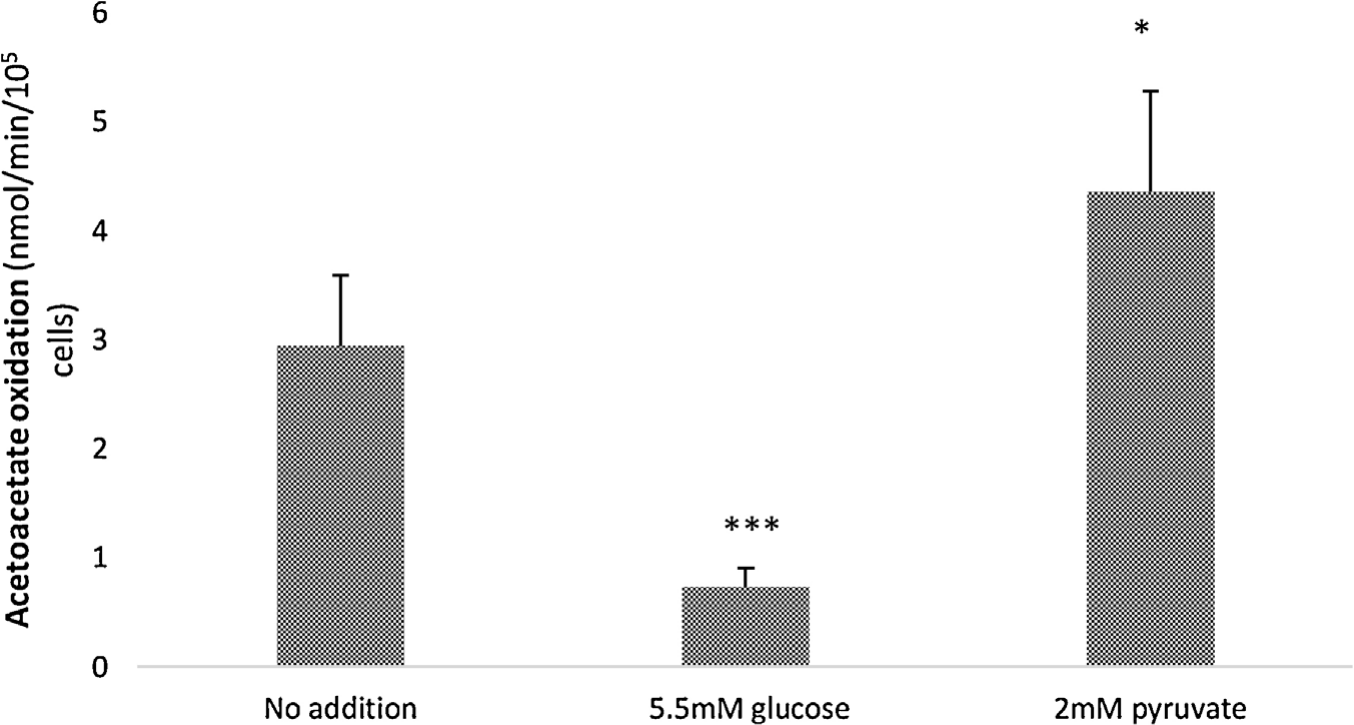
Oxidation of acetoacetate by hMSCs: effects of additional substrates. Rates of oxidation of 10 mM acetoacetate containing a trace of 3-^14^C-acetoacetate were measured in the presence of unlabelled 5.5 mM glucose or 2 mM pyruvate, where marked. Oxidation was measured in 24 well plates. Cells were passage 2, cultured under normoxic conditions. *: p < 0.05 and ***: p < 0.0001 compared with acetoacetate alone.

Data was analysed by single factor (Figs. 2 7a) or two factor (Fig. 1, 4 and 6) ANOVA.

## 3 Results

### 3.1 Oxidation of glucose, pyruvate, glutamine and acetoacetate

Oxidation rates for both glucose and acetoacetate rose with passage number for normoxic cultured cells (2-fold increase in glucose-oxidation rate and 2.6-fold increase in acetoacetate-oxidation rate) between passage 2 and passage 5 (p2-p5) whereas rates of oxidation of pyruvate and glutamine were not significantly different at the two passage numbers (Fig. 1A). Fig. 1 shows that acetoacetate was oxidised at a 27-fold higher rate than glucose at p2 and this rose to a 35-fold higher rate at p5. This increase was not seen for hypoxic-cultured cells, where the rate of oxidation of neither glucose nor acetoacetate was significantly different at p5 compared with p2, although that of pyruvate increased by 37%. Pyruvate and acetoacetate, which both feed directly into the mitochondrial TCA cycle, showed the highest rates of oxidation of all substrates for all culture conditions which indicates the functionality of the TCA cycle in these cells. When cells were cultured in the presence of 5 mM 3-hydroxybutyrate (3-HB) from passages 1–5, oxidation rates at passage 5 for normoxic cells were reduced by 4-fold for glucose and 3-fold for acetoacetate, giving an oxidation profile which more nearly resembled the 3-HB-free hypoxic condition than the 3-HB-free normoxic.

#### 3.1.1 Oxidation of acetoacetate: effects of additional substrates

Adding glucose to acetoacetate oxidation experiments reduced the rate of acetoacetate oxidation by 8-fold (Fig. 2) for normoxic cultured cells, suggesting either that the presence of glucose has a suppressing effect on acetoacetate utilisation or that pyruvate from glucose competes with acetyl CoA from acetoacetate for oxidation by the TCA cycle. However, addition of pyruvate resulted in a 1.5-fold increase in rates of acetoacetate oxidation so that competition seems unlikely. Indeed, it could be that the pyruvate substrate is carboxylated to oxaloacetate as a result of intramitochondrial pyruvate carboxylase activity (as well as being decarboxylated to acetyl Coenzyme A by pyruvate dehydrogenase) in these cells, so that the presence of pyruvate may maintain the capacity of the TCA cycle enabling continued acetoacetate oxidation. This effect was consistent for cells of all passage numbers (results not shown). This action would explain the increase in acetoacetate oxidation rates seen when pyruvate is present (Fig. 2). Addition of malate had no significant effect on rates of pyruvate oxidation (data not shown), indicating that carboxylation of pyruvate is not the only route for oxidative metabolism of this substrate. This may explain the difference observed between the effects of glucose and those of pyruvate on acetoacetate oxidation, despite the fact that both substrates would be expected to give rise to intramitochondrial pyruvate. The higher concentrations of intramitochondrial pyruvate achieved when 2 mM pyruvate is the substrate may lead to appreciable activity of pyruvate carboxylase, while lower concentrations of pyruvate from glucose may not.

#### 3.1.2 ATP production from substrate oxidation

**Fig. 3:**
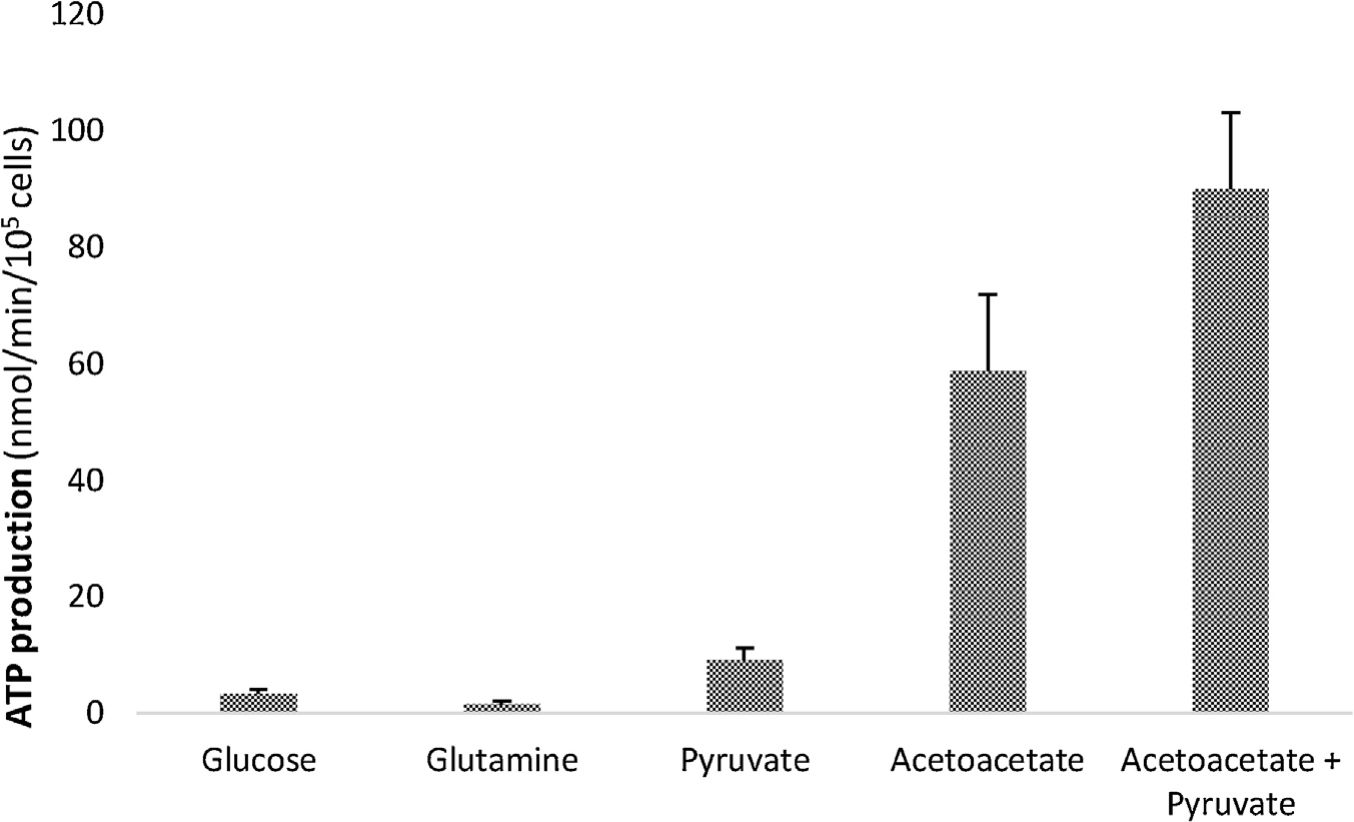
Calculated rates of ATP production by hMSCs with oxidative substrates. Rates of ATP production from individual substrates were calculated on the basis of mol ATP produced/mol substrate oxidised. Assuming full oxidation of each mole of substrate converted to CO_2.,_ it is calculated that 1 mol of glucose will produce a mean value of 31 mol ATP; glutamine: 20; pyruvate: 12.5; acetoacetate: 20 (refer to Materials and Methods for complete explanation). For calculations of ATP production from acetoacetate plus pyruvate, separate incubations were performed with 10 mM acetoacetate labelled with ^14^C-acetoacetate in the presence of unlabelled 2 mM pyruvate and with 2 mM pyruvate labelled with ^14^C-pyruvate in the presence of unlabelled 10 mM acetoacetate. Rates of ATP production from the labelled substrate in each incubation were calculated and the results summed. Cells were passage 2, cultured under normoxic conditions.

Calculated rates of ATP production demonstrate that acetoacetate is the single most efficient substrate for ATP production by these cells, when full oxidation of the substrate is assumed (Fig. 3). Neither glucose nor glutamine, the usual additions to tissue culture medium, were as productive of ATP (glucose produced 17 times less ATP than acetoacetate alone and glutamine produced 38 times less). The presence of pyruvate may increase the capacity of the TCA cycle and, therefore, the yield of ATP from acetoacetate, making this the most productive substrate combination for energy yield by these cells.

#### 3.1.3 Anaerobic metabolism of glucose: rates of glycolysis

**Fig. 4:**
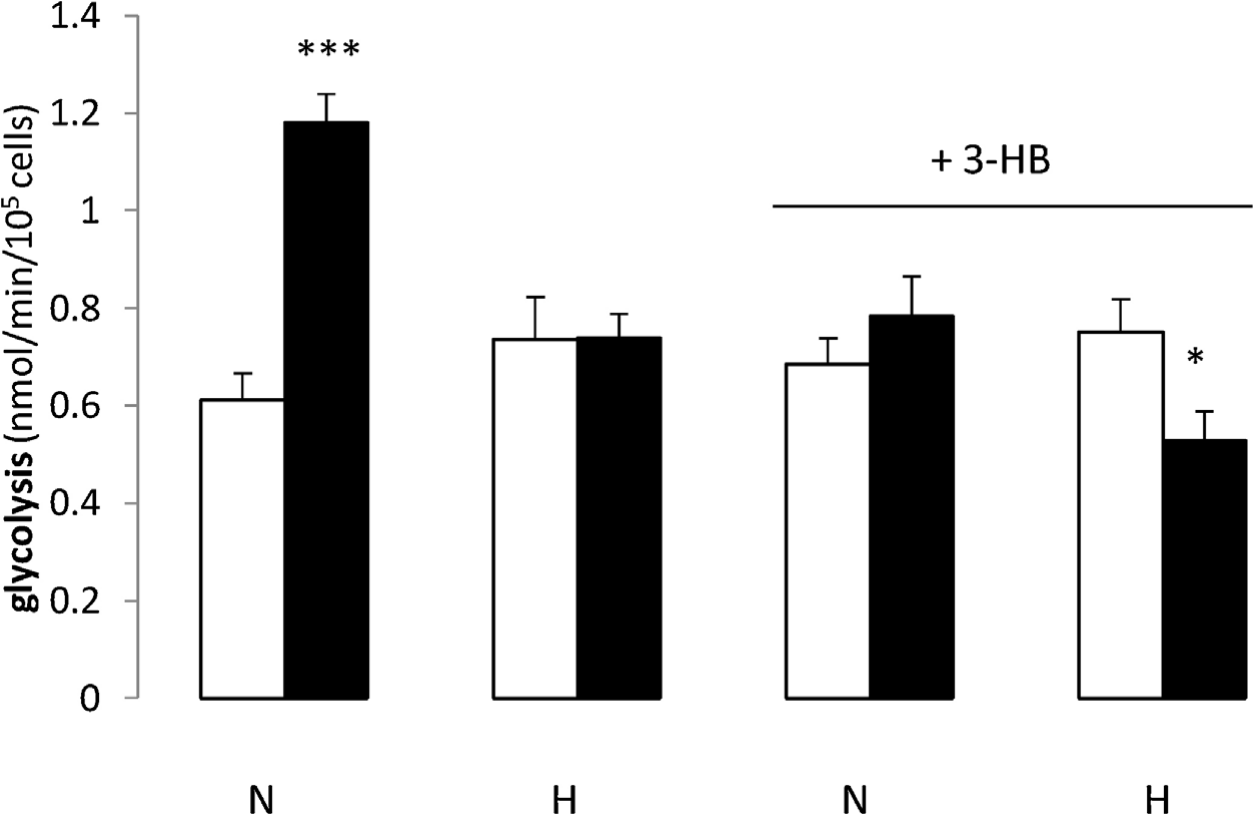
Rates of glycolysis by hMSCs at passages 2 and 5. Rates of glycolysis were measured for hMSCs at passages 2 (open columns) and 5 (solid columns) when cultured through passages 1–5 under normoxia (20% O_2_, N) or hypoxia (5% O_2_, H) with 5 mM 3-HB, where indicated. Glycolytic rates were measured in 24-well plates as the production of ^3^H_2_O from 5-^3^H-glucose by the method outlined in the Materials and Methods section. ***: p < 0.0001 compared with normoxic cells at passage 2; *p < 0.05 compared with cells cultured under the same conditions at passage 2.

Glycolytic rates were not significantly different for cells cultured under normoxic and hypoxic conditions at passage 2 (Fig. 4). However, more unexpected was the 1.9-fold rise in glycolytic rates seen for cells cultured under normoxia between passages 2 and 5. This effect was not seen for cells cultured under hypoxia and rates of glycolysis for hypoxic p5 cells were 37% lower than those for normoxic cultured cells. This mirrors the rise in oxidation rates seen for normoxic cultured cells (Fig. 1) and implies that energy yielding pathways are generally upregulated between the two passage numbers for cells cultured under 20% oxygen tension but not for cells cultured under 5% O_2_ tension. When the cells were cultured with 3-HB, glycolytic rates at p5 were unchanged relative to p2. For cells cultured under hypoxia with 3-HB, glycolytic rates were significantly decreased by passage 5 (1.4-fold). The presence of 3-HB thus seems to have a suppressing effect on the increase in energy yielding metabolism which follows from normoxic culture for both aerobic and anaerobic pathways (Fig. 1, Fig. 4). The energy profile of cells cultured in the presence of 3-HB and 20% O_2_ thus mimics that of cells cultured under hypoxic conditions.

#### 3.1.4 ATP production from anaerobic and aerobic metabolism of glucose

**Fig. 5:**
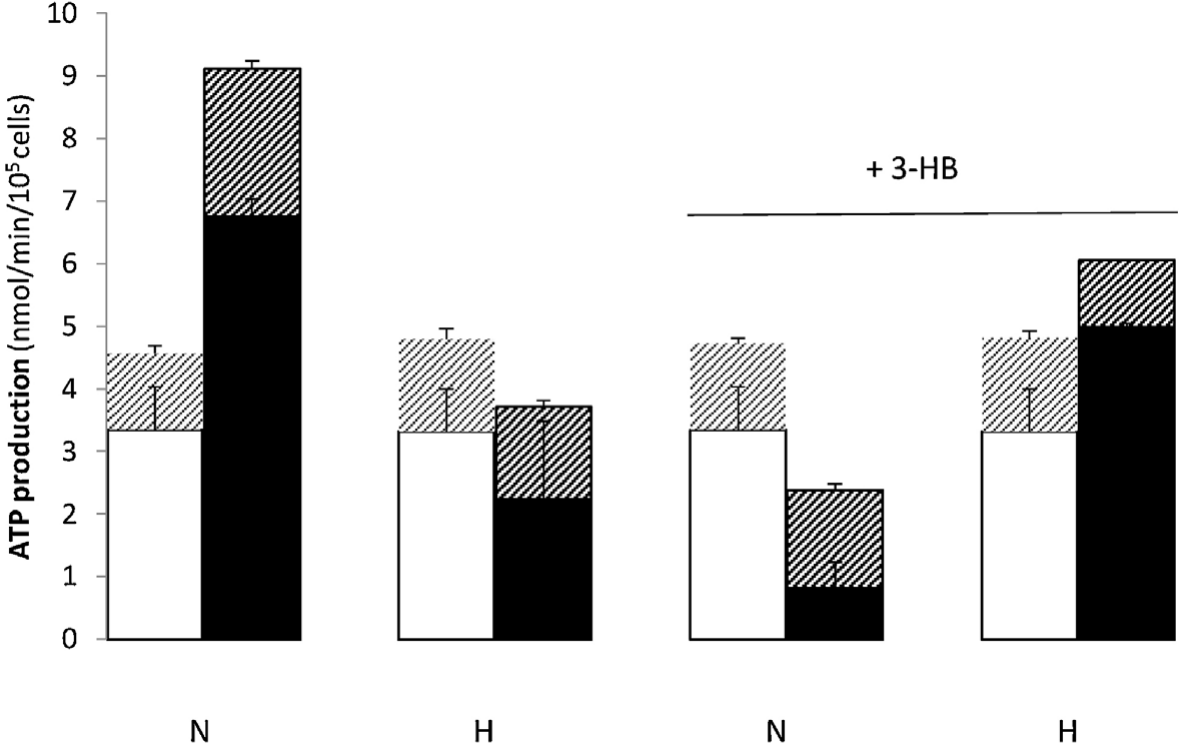
Calculated rates of ATP production by hMSCs from glycolysis and from full oxidation of glucose. Rates of ATP production were calculated as previously (refer to Materials and Methods for full explanation) from the anaerobic (glycolysis) and aerobic (oxidation) pathways for glucose metabolism. Cells were cultured under normoxic conditions (20% O_2_, N) or hypoxic (5% O_2_, H) with 5 mM 3-HB, where indicated, through passages 1–5. ATP production from full oxidation of ^14^C-labelled substrates is shown in the lower column of each data point and that from glycolysis is shown in the upper column. Measurements were made at passage 2 (open columns: oxidation; light patterning: glycolysis) and passage 5 (solid columns: oxidation; heavy patterning: glycolysis) for each culture condition. n ≥ 9.

Fig. 5 shows that normoxic cultured cells increase their calculated ATP production from both aerobic and anaerobic metabolism of glucose by a factor of 2 between passages 2 and 5. Interestingly, the proportion of ATP coming from each branch of metabolism remains constant (approximately 75% from glucose oxidation and 25% from glycolysis). Hypoxic cultured cells did not show the same increased rate of ATP generation and the inclusion of 3-HB in the culture medium of normoxic cells also reduced the passage-related increase in ATP generation producing a profile that resembles more closely that of hypoxic cultured cells than that of the normoxic. It would seem likely that the normoxic cultured cells increase their rate of ATP production either because they show increased rates of growth between passages 2 and 5 or because the cells are synthesising macromolecules in preparation for differentiation.

### 3.2 Growth rates of hypoxic- and normoxic-cultured cells between passages 2 and 5

**Fig. 6:**
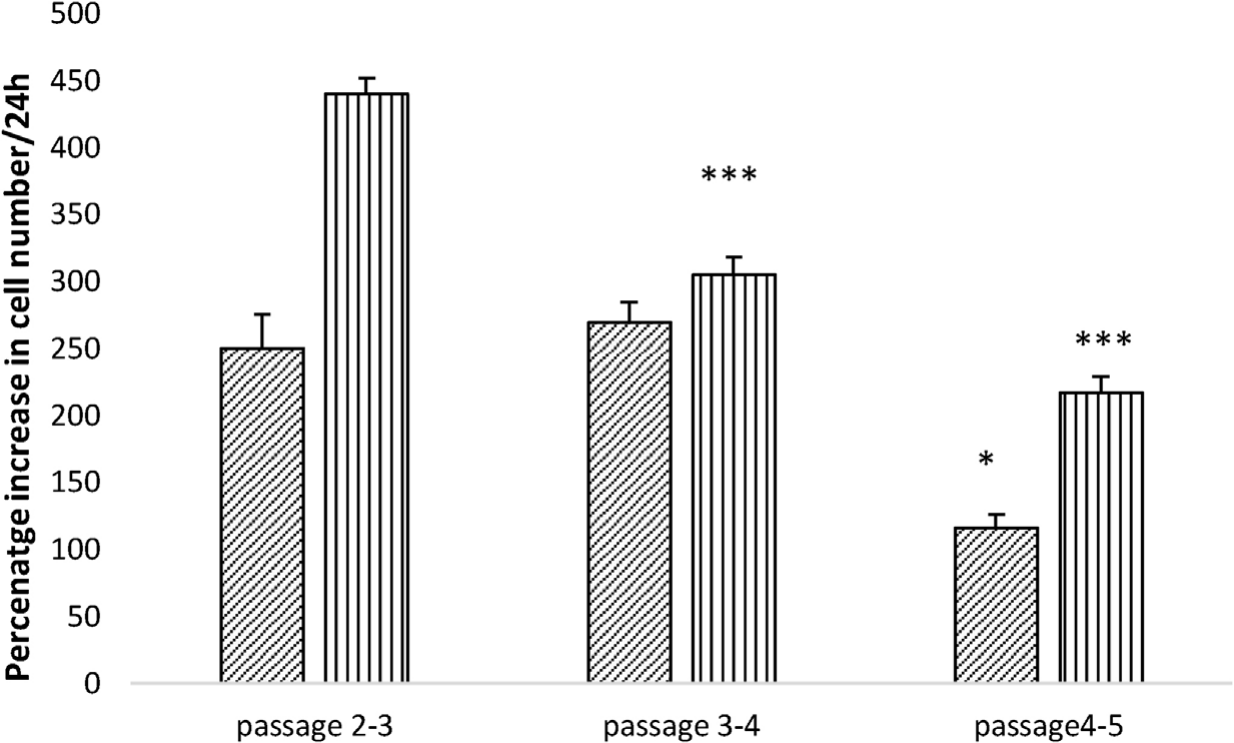
Growth rates of hMSCs with passage number. HMSCs were cultured under normoxic (20% O_2,_ cross-hatched) or hypoxic (5% O_2,_ vertical lines) conditions. Cells were passaged every 48 h and cell numbers counted by haemocytometer, using Trypan Blue to assess viability. n = 4 *: P < 0.05 compared with passage 2–3; *** p < 0.0001 compared with passage 2–3.

Fig. 6 demonstrates that growth rates declined with passage number for both normoxic and hypoxic culture conditions so that the cells were dividing at lower rates by passage 5 than at passage 2. This observation is true both for the normoxic cultured cells, which accelerate their rates of ATP production, and for the hypoxic cultured cells which do not.

#### 3.2.1 Production of mRNA for markers of stem-like, hMSC and differentiated status

Markers of MSCs, general stem-like status or differentiation did not change for normoxic cultured cells between passage 2 and 5 (Table S1). Interestingly, the only changes were seen during hypoxic culture, where the cells show increased production of mRNA for the general markers of the stem-like state, Oct4, c-myc and Sox2 and of the osteocyte-specific protein, osteopontin. None of the other markers of osteocytes show similar changes and Halfon et al. (2011) have reported that MSCs lose their osteogenic potential after passages 5–6, although these authors probably cultured their cells under normoxic conditions. Wang et al. (2013) grew MSCs from human umbilical cord for 30 passages and found that multipotent markers were still expressed. For the cells of the present study, there are no clear indications that differentiation is underway by passage 5.

#### 3.2.2 Differentiation of hMSCs along adipocytic, chondrocytic and osteocytic lineages

Cells grown under all conditions were assessed for their ability to differentiate at passage 6. All cells were successfully differentiated into adipocytes, chondrocytes and osteocytes and accumulation of intracellular fat, collagen and calcium were measured (Table S2). *Adipocyte differentiation*: intracellular fat accumulation was measured by Oil red O staining and differentiated cells showed a mean 30% increase over control cells which were cultured as normal and not subjected to the differentiation protocol. *Chondrocyte differentiation*: production of collagen (assessed by Sirius red/fast green staining) showed a mean 45% increase over control cells. *Osteocyte differentiation*: intracellular calcium accumulated to a mean 44% increase over control cells for cells cultured under hypoxic conditions but to a mean 26% increase for cells cultured under normoxia. There were no significant effects of culture conditions, except in the case of osteocyte differentiation stated above. Thus, hMSCs cultured under all conditions used in the present study retain the capacity to differentiate along all three expected lineages.

### 3.3 Production of ROS by hMSCs incubated with glucose and acetoacetate

**Fig. 7:**
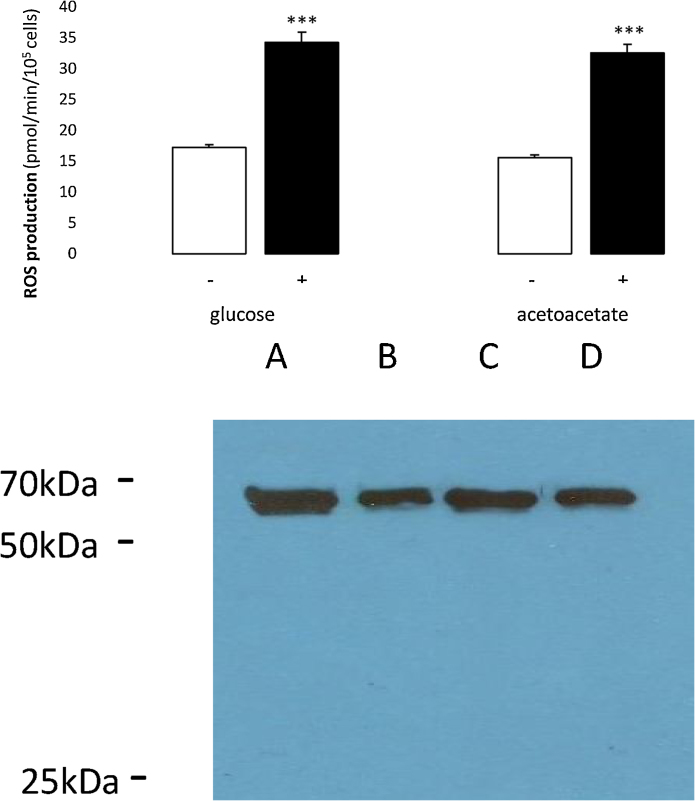
a Production of ROS by hMSCs with glucose and acetoacetate: effects of genipin. hMSCs were cultured under normoxic conditions and were passage 5 at the time of assay. For measurements in the presence of genipin, cells were pre-incubated for 24 h with 20 μM genipin. Cells were incubated for 90 min in 24 well plates in the presence of either 5.5 mM glucose or 10 mM acetoacetate before measurement of ROS. Values were calculated for 6 replicates. The first column of each pair represents production in the absence of genipin (−, open columns) and the second column, with genipin (+, solid columns). ***: P < 0.0001 for conditions with genipin compared with no addition. b Western Blot of hMSCs with anti-UCP2 antibody. Cell extracts were probed with anti-human UCP2 antibody and the probe detected using a secondary anti-rabbit antibody. Cells were cultured as follows: Lanes A and B under normoxic conditions and lanes C and D under hypoxic conditions. The presence of UCP2 protein was detected at passages 2 (lanes A and C) and 5 (lanes B and D).

It has been suggested that consumption of ketone bodies by cells may be a mechanism for the avoidance of oxidative stress (Fukao et al., 2004, Maalouf et al., 2009). In order to investigate whether substrate availability has an influence on oxidative stress in hMSCs, levels of ROS were measured after incubation with either a glucose or an acetoacetate substrate (Fig. 7a). If the ROS production is considered relative to rates of substrate oxidation, then each mole of glucose oxidised generated 0.076 mol of ROS whereas each mole of acetoacetate oxidised generated 0.0017 mol of ROS, a 45-fold difference. It has been suggested that the activity of UCP2 may influence the production of ROS (Negre-Salvayre et al., 1997, Arsenijevic et al., 2000). Effects of the specific UCP2 inhibitor, genipin (Zhang et al., 2006), indicate that this observation may be true for the hMSCs of the present study. The presence of genipin resulted in a 2 −fold increase in ROS production with either a glucose or an acetoacetate substrate. It has been shown by Echtay et al. (2002) that UCP2 functions to carry the superoxide anion. If this function is common to the cells of the present study, UCP2 may well act to reduce intramitochondrial ROS production, perhaps in a substrate-independent manner.

#### 3.3.1 Detection of human UCP2 by western blotting

The results of a Western blot using anti-human UCP2 antibody are shown in Fig. 7b. Cells cultured under all conditions show the presence of an approximately 66 kDa protein recognised by the antibody. The active UCP2 protein is a dimer composed of two subunits of approximately 32–35 kDa (reviewed by Ledesma et al., 2002). It has been shown for other members of the anion carrier superfamily that the two subunits may be covalently linked to form a functional unit (Trézéguet et al., 2000). Jekabsons et al. (2003) also observed an approximately 64 kDa protein recognised by anti-human UCP2 antibody in their preparations of purified human UCP2. No band corresponding to the monomer is seen in the cells of the present study at any passage number between 2 and 5 (results for intermediate passage numbers not shown).

## 4 Discussion

A salient characteristic of hMSC metabolism in the present study is the high rate of acetoacetate oxidation seen at all passage numbers and under both normoxic and hypoxic culture. Not only does this indicate that the cells have a functional TCA cycle of a higher capacity than would be deduced from their oxidation of glucose but it also highlights the effectiveness of acetoacetate as a potential energy yielding substrate. Indeed, the combination of acetoacetate plus pyruvate proved to be the most successful in terms of calculated ATP yield. This observation has obvious implications for the design of growth medium for the culture of hMSCs. Glucose and glutamine, the usual substrates included in standard growth medium, are not as effective in producing ATP and it could be that the design of a medium incorporating both a ketone body and pyruvate may stimulate energy production by these cells. Since any form of stem cell therapy must involve the harvesting and *in vitro* culture of stem cells, this is something which may bear further scrutiny. The significance of pyruvate for cultured cells has been indicated by the observation that cells do not thrive in a pyruvate-free medium but succumb to oxidative stress (O’Donnell-Tormey et al., 1987). Furthermore, Alt et al. (2005) have suggested that stem cells *in vivo* may take up pyruvate released by other cells in the stroma. The results of the present study support this suggestion as an effective energetic approach by these cells. The pyruvate may aid the cells’ metabolism by being carboxylated to oxaloacetate thus increasing the capacity of the TCA cycle and enabling increased oxidation of acetoacetate and other oxidative substrates.

Ketone bodies represent a fuel of intermediate and prolonged starvation, persisting at low levels in the blood in the fed state (approximately 0.1 mM) but rising after a fast of approximately 3–4 days in the human adult to levels that may be 70–80 times higher. Traditionally, ketone bodies have been regarded as a key fuel for neuronal tissues under these conditions. More recent reports have indicated that consumption of ketone bodies by cells, particularly neurones, may represent a way of reducing oxidative stress (Fukao et al., 2004, Maalouf et al., 2009) and it has been observed in general that stem cells tend to reside in an environment where there are low levels of ROS (Suda et al., 2011), which may be a strategy to aid their survival or their maintenance of the undifferentiated state. The current findings show that rates of ROS generation are 45-fold lower, on a mole ROS per mole substrate oxidised basis, with an acetoacetate substrate than they are with a glucose substrate. This may partly explain why oxidation of acetoacetate represents an appropriate metabolic strategy for these cells and may indicate a preference for an acetoacetate substrate when a range of substrates are available. Numerous recent reports have indicated a therapeutic role for a ketogenic diet, where a high ratio of fat: carbohydrate intake stimulates ketone body production so that plasma levels may reach 6–8 mM (Musa-Veloso et al., 2006). Such reports range from neuronal protection (Kashiwaya et al., 2012, Laeger et al., 2010) and neurogenesis (Lee et al., 2002a, Lee et al., 2002b)to more general protection against age-related damage (Paoli et al., 2013, Paoli et al., 2014). The reduction in ROS produced with a ketone body substrate may represent one of the mechanisms underlying these effects. Since hMSCs have a high rate of proliferation and a higher rate of ATP synthesis from acetoacetate, there is scope for speculation as to whether the ketogenic diet may have further applications in assisting with putative therapies based around stem cell transplantation, such as those for neurodegenerative disorders.

The effects of the UCP2 inhibitor, genipin, in increasing rates of ROS production 2-fold with glucose or acetoacetate, indicate the potential role for UCP2 in reducing ROS production in hMSCs. It may not be the case that UCP2 functions to uncouple electron transport from phosphorylation of ADP (reviewed by Pecqueur et al., 2009) but a role in substrate selection, causing a shift from carbohydrate to lipid metabolism, has been suggested (reviewed by Nedergaard and Cannon, 2003), in addition to a putative role in superoxide anion export from the mitochondrion (Echtay et al., 2002). From the results of the present study, a potential role for UCP2 in indicating the availability of acetoacetate and enabling its mitochondrial transport and oxidation is possible and ought to be investigated. Moreover, both Bouillaud (2009) and Zhang et al. (2011) have suggested that UCP2 may decrease the oxidation rate of pyruvate, perhaps by shunting this substrate out of the mitochondrion (Zhang et al., 2011), thereby favouring glycolysis. We find that hMSCs oxidise little endogenous pyruvate from glucose compared with their capacity for oxidising exogenous pyruvate, which would support this view.

Normoxic culture of hMSCs in the present study results in a general increase in flux through energy yielding pathways between passage 2 and 5. This includes an increase of 1.9–fold in glycolytic flux and a 2–fold increase in rates of glucose-oxidation and 2.6–fold of acetoacetate. The reasons for this increased flux are not clear since they seem to be unrelated to either growth rate (which declines between passage 2 and 5) or to the onset of differentiation (shown by non-significant changes in mRNA levels for key markers over the same time-scale). This effect appears to be related to the oxygen tension since it is not reproduced by the cells cultured under hypoxic conditions, although cells cultured under normoxia with 3-HB also fail to show an increase in energy generation. One further possibility is that there are changes in expression or activity of UCP2 over this time-scale, changing the cells’ substrate- and pathway-preferences and also perhaps changing anion transport activity. Western blots show that UCP2 is present at both passage 2 and passage 5. The possibility that the presence of oxygen at a 20% tension during long-term culture may alter rates of electron transport and production of ROS should be a subject for investigation. If this phenomenon is shown to be real, it would have implications for the long-term culture of stem cells under normoxic conditions for therapeutic or research purposes since any prolonged increase in ROS production may result in damage to DNA and higher rates of mutation and may account for the accumulation of genomic mutations observed by Wang et al. (2013) during long-term culture.

## 5 Conclusions

The consistent production of more ATP from oxidative pathways than from glycolysis by hMSCs indicated an active oxidative metabolism of high capacity. Thus, the occupancy of hypoxic niches by these cells does not curtail their oxidative capacity. The strong preference for the acetoacetate substrate shown by hMSCs, which may be necessary to reduce generation of ROS, means that this substrate should be considered for inclusion in growth medium. Up-regulation of both aerobic and anaerobic energy-yielding pathways when hMSCs were cultured in the presence of 20% O_2_ indicates that metabolic changes occur during the standard culture of these cells which may have an impact on the cells’ suitability for therapy.

## Author contributions

MB had conceptual control of the overall project, devised and performed all experiments contributing to Fig. 1, Fig. 2, Fig. 3, Fig. 4, Fig. 5, Fig. 6, Fig. 7 and Table S2 and wrote the manuscript. CL performed the experiments presented in Table S1. CvdB had conceptual input into the project, proposing the experiments presented in Fig. 1 and facilitated the supply of materials. RC advised on experimental design for Fig. 1, provided facilities and assisted with the preparation of the manuscript. KC provided facilities. CC advised on experiments presented in Table S1, suggested experiments presented in Table S2 and provided facilities.
