# Supplementary material for: Acetoacetate is a more efficient energy-yielding substrate for human mesenchymal stem cells than glucose and generates fewer reactive oxygen species
Source: Int J Biochem Cell Biol. 2017 Jul;88:75–83. doi: 10.1016/j.biocel.2017.05.007 (PMC5497396; doi:10.1016/j.biocel.2017.05.007)
Supplement: Supplementary file 1 [file mmc1.docx]

**Supplementary Information**

**Measurements of mRNA for marker proteins by qPCR (Table S1)**

Expression of marker proteins (MSC markers : CD73, CD105, Stro1, CD44, CD146, CD90; markers of pluripotency : Nanog, Oct4, cMyc, Sox2, TERT, PPARγ; markers of differentiated lineages : Osteopontin, BMP2, CD45, CD11b, CD14, Notch1) was assessed by qPCR. Cells of the appropriate passage number were pelleted and RNA extracted by anion exchange (Qiagen RNeasy Mini kit). The purity was assessed and the RNA quantified by nanospectrophotometer (Nanodrop Lite spectrophotometer). Synthesis of cDNA was achieved using an Applied Biosystems High Capacity cDNA Reverse Transcription Kit. Forward and reverse primers used to amplify target loci were, respectively : CD73 : CGCAACAAT GGCACAATTAC; CTCGACACTTGGTGCAAGA (Rada et al. 2011); CD105 : TCCTCCCAAGGACACTTGTA; CGCCTCATTGCTGATCATAC (Rada et al. 2011); Stro1 : GAAGCTAAAGTGGATTCAGGAGTA;TAAGCAGGGGACCATTACA (Rada et al., 2011); CD44 : GTTGCCAAACCACTGTTCCT; CATTCAAATCCGGAATGCT (Rada et al. 2011); CD146 : CCAAGGCAACCTCAGCCATGTC; CTCGACTCCACAGTCTCCCACGACT (Furstenberger et al. 2005); CD90 : GACAGCCTGAGAGGGTCTTG; CCCAGTGAAGATGCAGGTTT (Li et al, 2013); Nanog : ACCAGAACTGTGTGTTCTCTTCCACC; CCATTGCTATTCTTCGGCCAGTTG (Wilson et al. , 2010); Oct4 : GCTCGAGAAGGATGTGGTC; ATCCTCTCGTTGTGCATAGTCG (Liu et al. 2011); cMyc : CGTCTCCACACATCAGCACAA; TCTTGGCAGCAGGATAGTCCTT (Liu et al, 2011); Sox2 : CACTGTCCCTCTCACACATG; CCCATTTCCCTCGTTTTTCTT (Liu et al., 2011); TERT : GAGCTGACGTGGAAGATGAG; CTTCAAGTGCTGTCTCTGATTCCAATG (Friedrich et al, 2004); PPARγ : GGCTTCATGACAAGGGAGTTTC; AACTCAAACTTGGGCTCCATAAG (Zhang et al. 2007) ; Osteopontin : CCCACAGACCCTTCCAAGTA; GGGGACAACTGGAGTGAAAA (Rada et al., 2011); BMP2 : TCCTCTCATCAGCCATTTGTCCTTTC; AGTTACTACACATTCTTCATAG (Pera et al., 2004); CD45 : ACGAAGCTCTTAGCGTCAGG; CTCTCGGGTGGAGTCTTCTG (Li et al, 2013); CD11b : AGCCCAAGATCACATG; TGCAGAAGCATAACCC (Zhou et al., 2005); CD14 : GACTTATCGACCATGGAGCG; CCAGTAGCTGAGCAGGAACC (Pilz et al., 2011); Notch1 : GACTATGCCTGCAGCTGTGCC; GGCTGCAGGGCACGTAGG (Okumoto et al, 2003). cDNA was amplified during 40 cycles of PCR and quantified by Syb R-based fluorescence (Primer Design Precision FAST qPCR Mastermix). Gene expression at passage 5 compared with passage 2 was quantified by the 2^-ΔΔCT^ method (Livak and Schmittgen, 2001) relative to housekeeping genes (GAPDH; RNApolII;) at the same passage numbers.

**Table S1 : changes in mRNA-production for marker proteins at passage 5 compared with passage 2**

|  | **Fold change in mRNA** |  |
| --- | --- | --- |
| **Marker** | **Normoxic** | **Hypoxic** |
| **CD73** | 9.2 ± 0.9 | 1.000 ± 0.005 |
| **CD105** | 9.40 ± 0.89 | 0.500 ± 0.005 |
| **CD44** | 1.000 ± 0.005 | 30.0 ± 2.1 |
| **CD146** | 0.100 ± 0.005 | 0.100 ± 0.005 |
| **CD90** | 0.100 ± 0.005 | 0.100 ± 0.005 |
| **Stro1** | 0.100 ± 0.005 | 0.100 ± 0.005 |
| **Nanog** | 0.130 ± 0.004 | 1.600 ± 0.009 |
| **Oct4** | 4.9 ± 0.5 | 119 ± 8 |
| **cMyc** | 1.200 ± 0.005 | 60 ± 3 |
| **Sox2** | 0.810 ± 0.008 | 111 ± 4 |
| **TERT** | 0.044 ± 0.002 | 7.0 ± 0.4 |
| **PPARγ** | 0.46 ± 0.01 | 0.470 ± 0.003 |
| **Osteopontin** | 1.000 ± 0.005 | 239 ± 5 |
| **BMP2** | 1.000 ± 0.005 | 1.000 ± 0.005 |
| **CD45** | 0.93 ± 0.04 | 32 ± 1 |
| **CD11b** | 3.00 ± 0.05 | 1.000 ± 0.005 |
| **Notch1** | 2.60 ± 0.04 | 24 ± 2 |

Measurements of mRNA-synthesis were made by qPCR and values are expressed as the fold change at passage 5 relative to passage 2 (calculated by the 2^-∆∆CT^ method) when hMSCs were grown under conditions of either normoxia (20% O_2_) or hypoxia (5% O_2_). Mesenchymal stem cell markers (CD105, CD73, CD44); general markers of pluripotency (Nanog, Oct4, cMyc, Sox2, TERT, PPARϒ) and of differentiated cells (Osteopontin, BMP2, CD45, CD11b, Notch1) are shown.

**Differentiation of hMSCs (Table S2)**

Cells at passage 6 were seeded at a density of 2x10^5^ (adipogenesis and chondrogenesis) or 3x10^4^ (osteogenesis) into wells of a 6 well tissue culture plate. Kits for differentiation along adipocytic, chondrocytic and osteocytic lineages were obtained from Lonza and accompanying protocols followed. Early stage characteristics of differentiation were then assessed. Adipocytes were assayed for fat accumulation after 18 days from the onset of the differentiation protocol. Cells were fixed by incubating for 10 minutes in 10% formalin, followed by 60 minutes in fresh 10% formalin and washing with 60% isopropanol. 5mM Oil red O in 60% isopropanol (filtered), 1ml per well, was added to dry wells and left for 10 minutes before washing four times with water. Oil red O was eluted with 1ml 100% isopropanol per well and the optical density measured at 500nm. Chondrocytes were assayed for collagen accumulation (after 21 days from the onset of the differentiation protocol) using Sirius red/fast green collagen staining kit purchased from Amsbio. Osteocytes were assayed for calcium accumulation (21 days after the onset of the differentiation protocol) by the method of Jager et al, 2005. In each case, values were compared with those of control cells which had been cultured under normal growth conditions without being exposed to differentiation protocols.

**Table S2 : Measurements of fat, calcium and collagen for hMSCs differentiated along adipocytic, osteogenic or chondrocytic lineages**

**A : Absorbance at 500nm for cells stained with oil red O to measure triacylglycerol and cholesteryl ester accumulation**

| Absorbance of eluate at 500nm/culture condition | Adipocyte-differentiated cells | Control cells | % increase over control |
| --- | --- | --- | --- |
| Normoxia | *** 0.556 ± 0.001 | 0.473 ± 0.003 | 18 |
| Hypoxia | *** 0.638 ± 0.001 | 0.510 ± 0.003 | 25 |
| Normoxia + 5mM 3-HB | *** 0.698 ±0.005 | 0.520 ± 0.006 | 34 |
| Hypoxia + 5mM 3-HB | *** 0.727 ± 0.001 | 0.570 ± 0.001 | 27 |

Cells were inoculated at a density of 2x10^5^ cells/well into 6 well plates and the adipocyte differentiation protocol followed (refer to Experimental Procedures for full protocol). After 18 days, medium was removed from wells and cells stained with oil red O. The dye was eluted from the cells and the absorbance of the eluate read at 500 nm.

**B : Absorbance at 577nm for cells treated with cresopthalein to measure Ca^2+^ accumulation**

| Absorbance at 577nm/culture condition | Osteocyte-differentiated cells | Control cells | % increase over control |
| --- | --- | --- | --- |
| Normoxia | ** 0.385 ± 0.005 | 0.310 ± 0.003 | 24 |
| Hypoxia | *** 0.553 ± 0.009 | 0.376 ± 0.007 | 47 |
| Normoxia + 5mM 3-HB | *** 0.375 ± 0.003 | 0.310 ± 0.001 | 21 |
| Hypoxia + 5mM 3-HB | *** 0.495 ± 0.007 | 0.363 ± 0.003 | 36 |

Cells were inoculated at a density of 3x10^4^ cells/well into 6 well plates and the osteocyte differentiation protocol followed (refer to Experimental Procedures for full protocol). After 21 days, cells were incubated with 0.6M HCl for 24 hours before calcium per well was measured by complexing with cresopthalein and measuring absorbance at 577nm.

**C : Absorbance at 540nm for cells treated with Sirius red/fast green dye to measure collagen accumulation**

| Absorbance at 540nm/culture condition | Chondrocyte-differentiated cells | Control cells | % increase over controls |
| --- | --- | --- | --- |
| Normoxia | ** 0.410 ± 0.020 | 0.293 ± 0.004 | 40 |
| Hypoxia | *** 0.967 ± 0.012 | 0.643 ± 0.012 | 50 |
| Normoxia + 5mM 3-HB | *** 0.887 ± 0.020 | 0.319 ± 0.004 | 178 |
| Hypoxia + 5mM 3-HB | ** 0.806 ± 0.024 | 0.556 ± 0.008 | 45 |

Cells were inoculated at a density of 2x10^5^ cells/well into 6 well plates and the chondrocyte-differentiation protocol followed (refer to Experimental Procedures for full protocol). After 21 days, cells were fixed with Kahle fixative and stained with Sirius dye. Dye was extracted and the absorbance read at 540nm.

n = 3

** p<0.004; *** p<0.0001

**References to supplemental information**

Rada,T., Reis, R.L. and Gomes, M.E. (2011) Distinct stem cell subpopulations isolated from human adipose tissue exhibit different chondrogenic and osteogenic differentiation potential Stem Cell Rev. 7(1) 64-76.

Furstenberger, G., von Moos, R., Senn, H.J. and Boneberg, E.M. (2005) Real-time PCR of CD146 mRNA in peripheral blood enables the relative quantification of circulating endothelial cells and is an indicator of angiogenesis Br. J. Cancer 93 (7) 793-8.

Li, J., Xin, J., Zhang, L., Wu, J., Jiang, L., Zhou, Q., Li, J., Guo, J., Cao, H. and Li, L. (2013) Human hepatic progenitor cells express hematopoietic cell markers CD45 and CD109 Int. J. Med. Sci. 11(1) 65-79.

Wilson, K.D., Venkatasubrahmanyam, S., Fu, J.D., Sun, N., Abilez, O.J., Baugh, J.J., Jia, F., Ghosh, Z., Li, R.A., Butte, A.J. and Wu, J.C. (2010) Dynamic microRNA expression programs during cardiac differentiation of human embryonic stem cells: role for miR-499 Circ. Cardiovasc. Genet. 3(5) 426-35.

Liu, L., Wei, X., Ling, J., Wu, L. and Xiao, Y. (2011) Expression pattern of Oct-4, Sox2, and c-Myc in the primary culture of human dental pulp derived cells. J. Endod. 37 (4) 466-72.

Friedrich, M.G., Weisenberger, D.J.,, Cheng, J.C., Chandrasoma, S., Siegmund, K.D., Gonzalgo, M.L., Toma, M.I., Huland, H., Yoo, C., Tsai, Y.C., Nichols, P.W., Bochner ,B.H., Jones, P.A .and Liang, G. (2004) Detection of methylated apoptosis-associated genes in urine sediments of bladder cancer patients Clin. Cancer Res. 10(22) 7457-65.

Zhang, Y,, Ba, Y., Liu, C., Sun, G., Ding, L., Gao, S., Hao, J., Yu, Z., Zhang, J., Zen, K., Tong, Z., Xiang, Y. and Zhang, C.Y. (2007) PGC-1alpha induces apoptosis in human epithelial ovarian cancer cells through a PPARgamma-dependent pathway Cell Res. 17(4) 363-73.

Pera, M.F., Andrade, J., Houssami, S., Reubinoff, B., Trounson, A., Stanley, E.G., Ward-van Oostwaard, D., Mummery, C. (2004) Regulation of human embryonic stem cell differentiation by BMP-2 and its antagonist noggin J. Cell Sci. 117(Pt 7) 1269-80.

Zhou, X., Gao, X.P., Fan, J., Liu, Q., Anwar, K.N., Frey, R.S. and Malik, A.B. (2005) LPS activation of Toll-like receptor 4 signals CD11b/CD18 expression in neutrophils Am. J. Physiol. Lung Cell Mol. Physiol. 288(4) L655-62.

Pilz, G.A., Braun, J., Ulrich, C., Felka, T., Warstat, K., Ruh, M., Schewe, B., Abele, H., Larb,i A. and Aicher, W.K. (2011) Human mesenchymal stromal cells express CD14 cross-reactive epitopes Cytometry A. 79(8) 635-45.

Okumoto, K., Saito, T., Hattori, E., Ito, J.I., Adachi, T., Takeda, T., Sugahara, K., Watanabe, H., Saito, K., Togashi, H. and Kawata, S. (2003) Differentiation of bone marrow cells into cells that express liver-specific genes in vitro: implication of the Notch signals in differentiation Biochem. Biophys. Res. Commun.304(4) 691-5.

Jager, M., Feser, T., Denck, H. and Kraupse, R. (2005) Proliferation and osteogenic differentiation of mesenchymal stem cells cultures onto three different polymers in vitro Annals Biomed. Engineering **33** (10) 1319-32.
